# Nature of, and responses to key sexual and reproductive health challenges for adolescents in urban slums in sub-Saharan Africa: a scoping review

**DOI:** 10.1186/s12978-020-00998-5

**Published:** 2020-09-30

**Authors:** Yohannes Dibaba Wado, Martin Bangha, Caroline W. Kabiru, Garumma T. Feyissa

**Affiliations:** 1 African Population and Health Research Center, APHRC Campus, Manga Close, P.O. Box 10787-00100, Nairobi, Kenya; 2 Dornsife School of Public Health, Drexel University, Philadelphia, USA

**Keywords:** Slums, SRHR, Adolescents, Scoping review, SSA

## Abstract

**Background:**

Addressing adolescents’ sexual and reproductive health and rights (SRHR) requires an understanding of the socio-cultural and spatial settings within which they live. One setting of particular importance is the informal settlements or ‘slums’ that are gradually dominating the urban space. We undertook a scoping review and synthesis of existing evidence on adolescent SRHR in slums in sub-Saharan Africa (SSA) focusing on the characteristics and nature of existing evidence.

**Methods:**

The scoping review was conducted based on Arksey and O’Malley framework and in accordance with the guidance on scoping reviews from the Joanna Briggs Institute (JBI) and using PRISMA reporting guidelines for scoping reviews. A comprehensive search was undertaken in PubMed, POPLINE, African Journals Online (AJOL), Bioline International and Google Scholar. The search was confined to studies published in peer reviewed journals and reports published online between January 2000 and May 2019. Studies were included in the review if they addressed SRHR issues among adolescents living in urban slums in SSA.

**Results:**

The review included a total of 54 studies. The majority (79.5%) of studies were quantitative. The bulk of studies (85.2%) were observational studies with only eight intervention studies. While half (27) of the studies focused exclusively on adolescents (10–19 years), 12 studies combined adolescents with other young people (10–24 years). The studies were skewed towards sexual behavior (44%) and HIV/AIDS (43%) with very few studies focusing on other SRHR issues such as contraception, abortion, gender-based violence and sexually transmitted infections (STIs) other than HIV. Most of the studies highlighted the significantly higher risks for poor SRHR outcomes among adolescents in slums as compared to their peers in other settlements.

**Conclusion:**

Young people growing up in slums face tremendous challenges in relation to their SRHR needs resulting in poor outcomes such as early and unintended pregnancy, STIs, and sexual violence. The results of this review point to several potential target areas for programming, policy, and research aimed at improved adolescent SRHR in slums in SSA.

## Plain English summary

Addressing adolescents’ sexual and reproductive health and rights (SRHR) requires an understanding of the socio-cultural and spatial settings within which they live. This is critical in the current context where informal settlements or ‘slums’ are gradually dominating the rapidly expanding urban space. In recognition of the unique challenges, this review highlights existing evidence on adolescent SRHR in slums in sub-Saharan Africa (SSA) with particular attention to available policy responses.

Following standard guidance on scoping reviews from the Joanna Briggs Institute (JBI) and using PRISMA reporting guidelines, data bases such as PubMed, POPLINE, African Journals Online (AJOL), Bioline and Google Scholar were searched. We confined the search to studies published in peer reviewed articles and online reports published between January 2000 and May 2019 and found a total of 54 eligible studies. Half of these studies focused exclusively on adolescents (10–19 years), while the other combined adolescents with other young people.

Results highlight significant risks for poor SRHR outcomes among adolescents in slums as compared to their peers in other settlements. Most studies examined sexual behavior (44%) and HIV/AIDS (43%) while SRHR issues such as contraception, abortion and gender-based violence were rarely considered. Overall, the findings show that young people growing up in slums face tremendous challenges in relation to their SRHR needs resulting in poor outcomes such as early and unintended pregnancy, STIs, and sexual violence.

The results point to several potential target areas for programming, policy, and research aimed at improved adolescent SRHR in slums in SSA.

## Introduction

Globally, adolescents (ages 10–19 years) are a significant demographic block. In sub-Saharan Africa (SSA), adolescents account for more than 23% (or about 250 million) of the total population – a figure that is projected to increase rapidly in near future [1]. Adolescence is associated with physical, emotional and social changes that can increase vulnerability to poor sexual and reproductive health and rights (SRHR) outcomes and other risks associated with behavior change [2, 3].

Adolescents in low- and middle-income countries (LMICs) face tremendous challenges in relation to their SRHR needs. These include lack of access to SRHR information and services; lack of awareness about puberty, sexuality, and basic human rights; poverty; and inequitable gender norms that increase vulnerability to poor SRHR outcomes [4, 5]. Although adolescent SRHR is gaining global research and programmatic attention [6, 7], much of the research and program work overlooks adolescents in the fast growing urban slums in LMICs.

High rates of urbanization in SSA [1] amidst poor economic performance and weak governance has resulted in the growth of informal settlements, commonly referred to as slums. Currently, over half (55%) of urban dwellers in SSA reside in slums or slum-like environments that are characterized by dire poverty [8, 9]. Housing structures are temporary and are constructed from mud, iron sheets, cardboard boxes and polythene and plastic sheet tents. They are often located in undesirable parts of the city, such as steep hillsides, riverbanks or industrial areas [10]. The settlements are unregulated and unplanned and are thus characterized by overcrowding, poor sanitation, insecurity and poor access to social amenities. Because of their informal nature, government authorities are reluctant to provide social amenities and services such as schools, roads, and healthcare facilities. As a consequence, residents of slums have poorer health and socio-economic outcomes [10–12].

Urban slum residence creates a confluence of factors that place adolescents at heightened risk of poor SRHR outcomes [9]. For example, studies comparing SRHR outcomes between slum dwellers and non-slum dwellers in Nairobi show that slum residents are at greater risk for HIV infection, risky sexual behavior, early childbearing and maternal mortality [13–16]. Similarly, a study in Lagos, Nigeria shows that maternal mortality rates observed for two slum were higher than the figure estimated for the Lagos State [16].

Recognizing the unique challenges of urban poverty is critical in understanding the drivers of adolescent SRHR outcomes in the slum settings in order to implement effective programs for this critical age group. This review is motivated by the urgent need to understand the drivers of poor SRHR outcomes in slum settlements in SSA and inform prevention efforts. Understanding their SRHR is important for designing programs to improve health, education and employment outcomes among young people that are necessary to achieve the demographic dividend. By providing a comprehensive overview of available research and evidence on adolescent SRHR issues in slums in SSA, this review lay groundwork for a research agenda to explore key knowledge gaps concerning the nature and determinants of SRHR challenges among adolescent slum dwellers, and to inform interventions to address them.

### Objective

The aims of the scoping review were to map and describe available research and evidence on adolescent SRHR in slums in SSA. Specifically, we describe the characteristics, scope, nature of existing evidence and knowledge gaps.

## Methods

We conducted a scoping review method to identify and synthesize evidence on adolescent SRHR in slums in SSA. The review was conducted based on Arksey and O’Malley framework [17] and in accordance with the guidance on scoping reviews from the Joanna Briggs Institute (JBI) [18] and using PRISMA reporting guidelines for scoping reviews [19, 20]. We adopted the five-step method outlined by Arksey and O’Malley (2005): (1) identifying the research question; (2) identifying relevant studies/literature; (3) selecting studies; (4) charting the data; and (5) collating, summarizing, and reporting results.

In line with JBI guidelines, we outlined inclusion criteria for the population, concept, and context as follows:

### Population

The population considered included adolescents/teenagers (ages 10–19 years), young people, general population in the slum areas.

### Concept

Sexual and reproductive health outcomes such as sexual behavior; pregnancy and contraceptive use; HIV/AIDS, gender-based violence (GBV) (physical and sexual violence, intimate partner violence, female genital mutilation and early marriages).

### Context

The review considered the SRHR of adolescents living in urban slums, informal settlements, inner cities or deprived neighborhoods in urban areas in SSA.

### Search strategy

Prior to study selection and data abstraction, a review framework was developed to guide the identification of potentially relevant literature documents. A comprehensive search using databases was undertaken to locate articles published in peer reviewed journals and reports published online. An initial limited search of PubMed was done and text words contained in the titles and abstracts of relevant articles, and the index terms used to describe the articles were used to develop a full search strategy. Following this initial search, we searched the following databases: PubMed, POPLINE, African Journals Online (AJOL) and Bioline International and Google Scholar. The POPLINE website was retired on September 1, 2019 after our search was completed. Different combinations of the following search terms were used: Slums, informal settlements, deprived neighborhoods, inner city, sub-Saharan Africa, teenage/adolescent/ young women/people, young adolescents, SRHR of young people, sexual behavior; pregnancy, contraceptive use; HIV/AIDS, GBV (physical and sexual violence, female genital mutilation and early marriages).

Only articles written in English language published between January 2000 and May 2019 were considered. We also limited our review to studies that included adolescent girls and boys (aged 10–19 years) in their sample. We also reviewed the bibliographies of studies from the database searches to identify additional articles. Citation searches were also utilized which yielded new studies. We used Google to search for grey literature.

### Study selection

Studies identified from the initial search underwent title and abstract screening. After a full-text review, data were extracted from all selected studies, including the year of publication, country and city of origin, the age and sex category of the study population, the design and type of study, whether it was a single or multi-site study, whether it was a single or multi-topic study, type of SRHR primary outcome. Studies that focused on general urbanization and/or urban poverty and health were reviewed separately and information on the substantive issue was extracted. Included papers were critically appraised using the Preferred Reporting Items for Systematic Reviews and Meta-Analyses—Extension for Scoping Review (PRIMSMA-ScR) guideline [20].

## Results

**Fig. 1:**
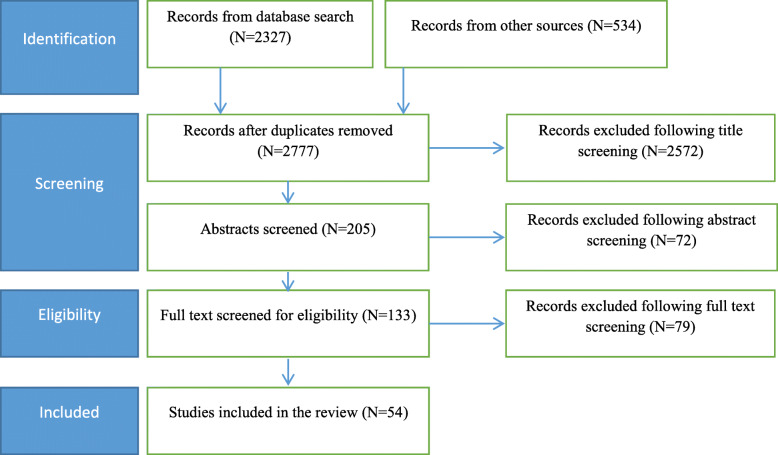
Study selection procedure for the scoping

The initial search yielded a total of 2861 studies. After removing duplicates, 2777 records were left for screening. Upon completion of title and abstract screening, 72 were excluded leaving 133 full-texts deemed potentially relevant for review. Subsequently, 54 documents fulfilled our eligibility criteria and were included in this review (Fig. 1).

The studies identified were reviewed and grouped according to the following categories: sexual behavior; pregnancy and contraceptive use; HIV/AIDs, GBV (physical and sexual violence, female genital mutilation and early marriages). These categories were subsequently used to structure the presentation of results in a systematic manner. It is worth noting that several studies addressed more than one category.

The studies encompassed a variety of study designs including cross-sectional studies, pre–post designs, randomized controlled trials, retrospective and prospective cohort studies and case–control studies (Table 1). We organize our results systematically, beginning with studies on urbanizations, slums and adolescent health in general, then we focus on specific substantive SRHR topics before turning to interventions on adolescent SRHR.

**Table 1:** characteristics of studies included in the review

Area of Focus	Number	%
Country		
Kenya	31	57.4%
South Africa	9	16.7%
Ethiopia	4	7.4%
Nigeria	3	5.4%
Uganda	2	3.7%
Zimbabwe	2	3.7%
Ghana	1	1.9%
Tanzania	1	1.9%
Malawi	1	1.9%
Design/ Method		
Quantitative	41	79.5
Qualitative	7	13.0%
Mixed method	6	11.1%
Type		
Observational	46	85.2%
Intervention/Implementation Research	8	14.8%
Population		
Girls	26	48.1
Boys and Girls	28	51.9
Age groups		
10–19	18	33.3
15–19	9	16.7
10–24	12	22.2
15–49	15	27.8
Period		
2000–2004	5	9.3
2005–2009	9	16.7
2010–2014	24	44.4
2015–2019	16	29.6
Total	**54**	**100**

### Characteristics of studies on adolescent SRHR in slums

Among the studies that focused on the substantive topic of adolescent SRHR in slums in SSA, more than half (*n* = 31; 57.4%) were based on slums in Nairobi, Kenya’s capital city (Viwandani, Korogocho and Kibera). Nine (16.7%) studies covered settlements in South Africa (Cape Town, Johannesburg and Durban), while four (7.4%) covered Ethiopia (Addis Ababa) and three, Nigeria (Ibadan). Two studies each were based on slums in Uganda (Kampala) and Zimbabwe (Harare) while one study covered slums in Ghana (Accra), Tanzania (Dar es Salaam) and Malawi (Blantyre).

Majority 41(79.5%) were quantitative studies while qualitative and mixed methods studies accounted for 13% (*n* = 7) and 11% (*n* = 6) respectively. The bulk of studies (*n* = 46; 85.2%) were observational, cross-sectional studies while 14.8% (*n* = 8) reported on interventions or implementation research.

In terms of study population, many of the studies reviewed did not strictly adhere to the World Health Organization (WHO) definition of adolescents (i.e., those aged 10–19 years). Rather, they combined adolescents with other age groups such as young people (10–24); youths (15–24 years) and women of reproductive age (15–49 years). About half of the studies (27) focused exclusively on adolescents (10–19 years). Twelve studies combined adolescents with other young people (10–24 years), while fifteen treated them as a category in the reproductive age group (15–49 years). For half of the studies, the respondents were girls or young women while the other half combined boys and girls.

It is evident that literature on the adolescent SRHR in slums in SSA has been growing steadily over the last two decades. Dividing the period under consideration into five-year intervals, we noted that 9.3% were conducted between 2000 and 2004. About 44% of the studies on adolescent SRHR in the slums in SSA were done between 2010 and 2014. Majority (74%) of studies were conducted over the last decade (from 2010 to date).

### Adolescent sexual behavior in slums

This review identified 24 studies (44%) that investigated sexual behavior among adolescent in the slums of SSA cities. The majority of these studies were quantitative, observational and from Nairobi City slums (Table 2). Most of these studies assessed “risky” sexual behavior, virtually to the exclusion of other sexual behavior among young people.

**Table 2:** Studies on adolescent sexual behavior

Study	Slum Name	City	Population	Age group	Study Type	Design	Sample size
Abebe, 2006	Teklehamanot	Addis Ababa	Girls & Boys	10–19	Obs.	Quant.	186
Adebola A. and Odutolu 2007	Not specified	Ibadan	Girls & Boys	15–24	Obs.	Quant.	1042
Amuyunzu-Nyamongo and Magadi 2006	Korogocho & Viwandani	Nairobi	Girls & Boys	13–50	Obs.	Qual.	40
Austrian et al. 2015	Kibera	Nairobi	Girls	18–25	Interv	Qual	128
Beguy, et al. 2013	Korogocho & Viwandani	Nairobi	Girls & Boys	12–22	Obs.	Mixed	4058
Beguy et al. 2009	Korogocho &Viwandani	Nairobi	Girls & Boys	12–19	Obs.	Quant.	2324
Carolina for Kibera 2007	Kibera	Nairobi	Girls	11–18	Interv	Quant	222
Dodoo, Zulu, and Ezeh 2007	Korogocho & Viwandani	Nairobi	Girls & Boys	15–49	Obs.	Mixed method	15,315
Erulkar and Ferede 2009	Not Specified	AA^a^,BD^b^ & Gondar	Girls	10–19	Obs.	Quant	1839
Erulkar et al. 2013	Not Specified	Addis Ababa	Girls	10–19	Interv	Quant	1172
Erulkar et al. 2004	Merkato & Kazanchis	Addis Ababa	Girls & Boys	10–19	Obs.	Quant.	1076
Kabiru et al. 2010	Korogocho & Viwandani	Nairobi	Girls & Boys	12–19	Obs.	Quant.	2134
Khoza et al. 2018	Hillbrow, Berea, & Yeoville	Johannesburg	Girls & Boys	16–18	Interv	Qual.	120
Marston et al. 2013	Korogocho & Viwandani	Nairobi	Girls & Boys	10–19	Obs.	Quant	1754
Mphatso Kamndaya et al. 2015	Mbayani and Mtopwa	Blantyre, Malawi	Girls & Boys	10–24	Obs.	Qual	60
Motsomi et al. 2016	Zandspruit	Johannesburg	Girls & Boys	15–19	Obs.	Qual	40
Mugisha and Zulu 2004	Korogocho & Viwandani	Nairobi	Girls & Boys	10–24	Obs.	Mixed method	Not Stated
Ndugwa et al. 2011	Korogocho & Viwandani	Nairobi	Girls & Boys	12–19	Obs.	Quant	1722
Ngom, Magadi, and Owuor 2003	Korogocho & Viwandani	Nairobi	Girls	10–19	Obs.	Quant	788
Okigbo et al. 2015	Korogocho & Viwandani	Nairobi	Girls & Boys	12–19	Obs.	Quant.	1927
Renzaho et al. 2017	Makindye and Nakawa	Kampala	Girls & Boys	10–24	Obs.	Quant	663
Sidze et al. 2015	Korogocho & Viwandani	Nairobi	Girls & Boys	10–24	Obs.	Quant	689
Ziraba et al. 2018	Korogocho & Viwandani	Nairobi	Girls	12–23	Obs.	Quant.	1390
Zulu, Dodoo, & Ezeh 2002	Korogocho & Viwandani	Nairobi	Girls & Boys	15–49	Obs.	Quant.	1645

Where: Obs. ➔Observational research; Interv➔ Intervention research, Qual➔ Qualitative; Quant➔ Quantitative, AA^a^-Addis Ababa BD ^b^– Bahir Dar

The sexual risk behaviors examined include early age sexual debut [11, 15, 21] unprotected sexual intercourse [15, 22, 23], multiple sexual partnership [11, 15, 24], transactional sex [11, 15, 25] and age-disparate sex [25, 26].

Several studies examined factors that influence adolescent SRHR through the lens of an ecological framework outlining individual, familial/relational, societal and structural level factors. The slum environment in these studies was conceptualized as a mediating variable that intensifies the effect of these factors to cause adverse SRHR outcomes.

Study results showed that risky sexual behavior is strongly shaped by individual and socio-structural forces. The individual factors highlighted include: poor knowledge or access to protection (condoms) [23, 27]. The extent to which adolescents are knowledgeable about protection and have access to them was noted to be a factor determining their use of condoms to prevent against pregnancy, HIV/AIDS and other STIs. Other individual level factors notably associated with risky sexual behavior among adolescents were alcohol, drug and substance use [28–30].

Several studies also reported that young people living in urban slums engage in sexual intercourse much earlier and/or transactional and age disparate sex [11, 14, 15, 28, 31]. Adolescents living in slums were found to be more likely to have multiple sexual partners and to report that their most recent sexual intercourse was unprotected than their peers living in wealthier households.

Parental factors such as co-residence and parental control were also highlighted by some studies [15, 25, 32–34]. These studies show that adolescents living in informal settlements are more likely to be staying on their own or with friends and thus lack formal parental control, which may expose them to riskier behaviors.

Other social factors identified include peer and partner influences that operate in a gendered context and affect adolescent boys and girls differently [15, 23, 25, 28]. Studies suggest that adolescents’ relationship dynamics are characterized by unequal decision-making between male and female partners with females having less control over their sexual lives. There is also poor communication about sexual matters by male and female adolescent sexual partners and hence a lack of preparation for or anticipation of intercourse [35].

### Adolescent pregnancy

The review identified 16 studies that investigated adolescent pregnancy. Majority (69%) of these studies were carried out in Nairobi. Two studies were conducted in Addis Ababa and one each in Kampala, Blantyre, and Johannesburg. The majority of these studies were observational and quantitative in type and design respectively. Only three studies were interventions or implementation research, and five were qualitative or mixed methods in nature.

Most of these studies examined the correlates of adolescent pregnancy in the slums (Table 3). Several studies looked at **individual behavior** correlates. These were: Low levels of knowledge on the menstruation cycle [15, 24, 32, 36]; low levels of knowledge on contraception or non-use of contraception [37, 38]; early sexual debut; multiple or frequent sexual partnership [11, 24, 36]; transactional and age-disparate sex [30, 32] and alcohol and drug use [28, 37, 39].

**Table 3:** Studies on adolescent pregnancy

Study	Slum Name	City	Population	Age group	Study Type	Design	Sample size
Abebe, 2006	Teklehimanot	Addis Ababa	Girls & Boys	10–19	Obs.	Quant	186
Austrian et al., 2015	Kibera	Nairobi	Girls	18–25	Interv	Qual	128
Austrian et al. 2015	Kibera	Nairobi	Girls	11–14	Interv	Quant	6000
Austrian et al. .2018	Kibera	Nairobi	Girls	11–14	Interv	Quant	6000
Beguy, Ndugwa, and Kabiru 2013	Korogocho & Viwandani	Nairobi	Girls	15–19	Obs.	Quant	897
Beguy, et al. 2013	Korogocho & Viwandani	Nairobi	Girls & Boys	12–22	Obs.	Mixed method	4058
Beguy et al. 2014	Korogocho & Viwandani	Nairobi, Kenya	Girls	10–24	Obs.	Quant	846
Brahmbhatt et al. 2014	Not specified	Johannesburg, Ibadan	Girls & Boys	15–19	Obs.	Quant	1112
Erulkar et al. 2004	Merkato & Kazanchis	Addis Ababa	Girls & Boys	10–19	Obs.	Quant	1076
Jayaweera et al. 2018	Not Specified	Nairobi	Girls	15–35	Obs.	Qual	71
Kabiru et al. 2010	Korogocho & Viwandani	Nairobi	Girls & Boys	12–19	Obs.	Quant	2134
Mphatso et al. 2015	Mbayani and Mtopwa	Blantyre	Girls & Boys	10–24	Obs.	Qual	60
Mumah et al. 2014	Korogocho & Viwandani	Nairobi	Girls	15–19	Obs.	Qual	31
Ngom, Magadi, and Owuor 2003	Korogocho & Viwandani	Nairobi	Girls	10–19	Obs.	Quant	788
Renzaho et al. 2017	Makindye and Nakawa	Kampala	Girls & Boys	10–24	Obs.	Quant	663
Zulu, Dodoo, and Ezeh 2002	Korogocho & Viwandani	Nairobi	Girls & Boys	15–49	Obs.	Quant	1645

Where: Obs.➔ Observational research; Interv➔ Intervention research, Qual➔ Qualitative; Quant➔ Quantitative

Other studies looked at socio-ecological factors associated with pregnancy. These were poverty [11, 15, 28, 37]; peer pressure [15, 36, 37]; lack of parental control [15, 33, 39, 40] and the physical environment [11, 13, 39].

### HIV/AIDS and other STIs

The review identified 23 studies that investigated HIV/AIDS in the slums of SSA cities. Some of these studies examined the prevalence and/or incidence of HIV/AIDS in the slums in comparison with other settlements. From these studies (Table 4), the prevalence of HIV was significantly higher in slums than other urban areas and other settlements. For example, in Nairobi the HIV prevalence among young people aged 15–19 years in informal urban settlements (15.2%) was relatively higher than in formal settlements (11.4%) [13]. Similarly, across 20 countries in SSA, it was reported that the urban poor have on average 19% *higher* odds of being HIV positive than their non-poor urban counterparts of similar background characteristics (age, gender, educational attainment, gender of household head and religion [41].

**Table 4:** Studies on HIV/AIDS

Study	Name of Slum	City	Population	Age group	Study Type	Design	Sample size
Abebe, 2006	Teklehimanot	Addis Ababa	Girls & Boys	10–19	Obs.	Quant	186
Adebola et al., 2007	NS	Ibadan	Girls & Boys	15–24	Obs.	Quant	1042
Adedimeji et al. 2008	Ita-Ege, Esu-Awele, Isale-Ijebu	Ibadan	Girls & Boys	15–24	Obs.	Quant	886
Austrian, et al., 2015	Kibera	Nairobi	Girls	18–25	Interv	Qual	128
Beguy, et al. 2013	Korogocho & Viwandani	Nairobi	Girls & Boys	12–22	Obs.	Mixed	4058
Dodoo et.al, 2007	Korogocho & Viwandani	Nairobi	Girls & Boys	15–49	Obs.	Mixed Method	15,315
Dunbar et al. 2010	NS	Harare	Girls	15–19	Interv	Quant	315
Erulkar et al. 2004	Merkato & Kazanchis	Addis Ababa	Girls & Boys	10–19	Obs.	Quant	1076
Erulkar et al. 2013	NS	Addis Ababa	Girls	10–19	Interv	Quant	1172
Gibbs et al. 2017	eThekwini	Durban	Girls & Boys	18–30	Interv	Mixed method	232
Greif et al., 2010	NS	Dar^a^, Kam^a^, Accra, Harare	Girls	15–49	Obs.	Quant	Not stated
Hall et al., 2006	Kibera	Nairobi	Girls	16–22	Interv	Quant	255
Henwood et al. 2016	Khayelitsha	Cape Town	Girls & Boys	12–25	Interv	Mixed method	60
Kamndaya et al. 2014	NS	South Africa	Girls & Boys	10–24	Obs.	Quant	530
Kabiru et al. 2010	Korogocho & Viwandani	Nairobi	Girls & Boys	12–19	Obs.	Quant	2134
Kabiru et al. 2011	Korogocho & Viwandani	Nairobi	Girls & Boys	12–22	Obs.	Quant	4028
Khoza et al. 2018	NS	Johannesburg	Girls & Boys	16–18	Interv	Qual	120
Madise et al. 2012	Korogocho & Viwandani	Nairobi	Girls & Boys	15–49	Obs.	Quant	5048
Mphatso Kamndaya et al. 2015	Mbayani and Mtopwa	Blantyre, Malawi	Girls & Boys	10–24	Obs.	Qual	60
Mugisha and Zulu 2004	Korogocho & Viwandani	Nairobi	Girls & Boys	10–24	Obs.	Mixed method	Not stated
Renzaho et al. 2017^a^	Makindye and Nakawa	Kampala	Girls & Boys	10–24	Obs.	Quant	663
Ziraba et al. 2018	Korogocho & Viwandani	Nairobi	Girls	12–23	Obs.	Quant	1390
Zulu, Dodoo, and Ezeh 2002	Korogocho & Viwandani	Nairobi	Girls & Boys	15–49	Obs.	Mixed method	1645

^a^Addressed HIV/AIDS and other STI;

Where: Obs.➔ Observational research; Interv➔ Intervention research, Qual➔ Qualitative; Quant➔ Quantitative; Dar ➔ Dares Salaam; Kam➔ Kampala; Har ➔ Harare; NS – not specified

HIV/AIDS is often transmitted through heterosexual intercourse and most studies in the slum settlements examined sexual behavior patterns that predispose adolescents to HIV infection [11, 13–15, 26, 36, 42]. It is important to note that due to the criminalization of same sex relations in SSA, an accurate estimate of the modes of HIV transmission is difficult. Several studies examined how poverty and related economic hardships contribute to risky sexual practices and may increase risk of infection [11, 14, 15, 28, 31, 43].

### Contraception and abortion

About a fifth of the studies examined contraceptive use: three of which investigated patterns of a full range of contraceptives [22, 42, 44], while six studies examined condoms use patterns for dual protection against pregnancies and HIV/AIDS [23, 28, 45–47]. These studies show that adolescents face many barriers in the use of contraceptive methods including lack of access, fear/embarrassment, cost and lack of knowledge. Consequently, the use of contraception among adolescents remains low leading to high levels of unintended pregnancies, unplanned births and unsafe abortion (Table 5).

**Table 5:** Studies on Contraception and Abortion

Study	Slum Name	City	Population	Age group	Study Type	Design	Sample size
	**Contraception/Family planning**						
Abebe 2006	Teklehaimanot	Addis Ababa	Girls & Boys	10–19	Obs.	Quant	186
Adebola et.al 2007	Not specified	Ibadan	Girls & Boys	15–24	Obs.	Quant	1042
Adedimeji et al. 2008	Ita-Ege, Esu-Awele, Isale-Ijebu	Ibadan	Girls & Boys	15–24	Obs.	Quant	886
Austrian et al. 2015	Kibera	Nairobi	Girls	11–14	Interv	Quant	6000
Austrian et al. 2018	Kibera	Nairobi	Girls	11–14	Interv	Quant	6000
Beguy, et al. 2013	Korogocho & Viwandani	Nairobi	Girls & Boys	12–22	Obs.	Mixed method	4058
Ochako et al. 2016	Korogocho & Viwandani	Nairobi	Girls	15–49	Obs.	Quant	1873
Renzaho et al. 2017	Makindye and Nakawa	Kampala	Girls & Boys	10–24	Obs.	Quant	663
Sidze et al. 2015	Korogocho & Viwandani	Nairobi	Girls & Boys	10–24	Obs.	Quant	689
Ziraba et al. 2018	Korogocho & Viwandani	Nairobi	Girls	12–23	Obs.	Quant	1390
	**Abortion**						
Jayaweera et al. 2018	Not Specified	Nairobi	Girls	15–35	Obs.	Qual	71
Kenya Human Rights Commission 2010	Korogocho	Nairobi	Girls	15–27	Obs.	Mixed method	65
Renzaho et al. 2017	Makindye and Nakawa	Kampala	Girls & Boys	10–24	Obs.	Quant	663

Where: Obs.➔ Observational research; Interv➔ Intervention research, Qual➔ Qualitative; Quant➔ Quantitative

Only three studies focused on abortion among adolescent girls [38, 42, 48]. These studies underscore unsafe abortion resulting from unintended pregnancies among adolescent girls as a maternal health issue of great concern in most SSA settings where abortion laws are restrictive and safe abortion is largely inaccessible. Therefore, many adolescents in need of pregnancy termination resort to unsafe abortion, which is associated with significant mortality and morbidity risks.

### Gender-based violence

This review identified nine (17%) studies that investigated GBV among adolescent (Table 6), most of which examined intimate partner physical and sexual violence [27, 29, 42, 49–51]. Very few studies focused on child marriage and female genital mutilation [52].

**Table 6:** Studies on Gender-Based Violence

Study	Slum name	City	Population	Age group	Study Type	Design	Sample size
**General Gender-Based Violence**							
Abuya et al. 2012	Not Specified	Nairobi	Girls	15–19	Obs.	Qual	10
Austrian et al. 2015	Kibera	Nairobi	Girls	11–14	Interv	Quant	6000
Austrian et al. 2018	Kibera	Nairobi	Girls	11–14	Interv	Quant	6000
Erulkar et al. 2013	Not Specified	Addis Ababa	Girls	10–19	Interv	Quant	1172
Gibbs et al. 2017	eThekwini	Durban	Girls & Boys	18–30	Interv	Mixed method	232
Mugisha and Zulu 2004	Korogocho & Viwandani	Nairobi	Girls & Boys	10–24	Obs.	Mixed method	Not stated
Renzaho et al. 2017	Makindye and Nakawa	Kampala	Girls & Boys	10–24	Obs.	Quant	663
Swart E. 2012	Kibera	Nairobi	Girls	18–30	Obs.	Quant	200
**Female Genital Mutilation**							
Mudege et al. 2012	Korogocho&Viwandani	Nairobi	Girls	12–24	Obs.	Quant	527

Where: Obs.➔ Observational research; Interv➔ Intervention research, Qual➔ Qualitative; Quant➔ Quantitative

The studies that examined the prevalence, causes and consequences of GBV suggest that it is common among adolescents in slum settlements with adolescent girls more likely to be affected than boys [29, 50–52]. Accordingly, the main driver of GBV may be traditional norms and cultural beliefs that men are more powerful than women and therefore should dominate or control women and their sexuality including sexual intercourse [50–52]. Other correlates include alcohol and drug use [29] and commercial and/or transactional sex [50]. The consequences of GBV highlighted include injury, pain, psychological distress and other mental health illnesses, STIs, including HIV/AIDS, and unintended pregnancy [47, 50, 51].

### Programs and interventions

The review identified nine interventions targeting adolescent SRHR outcomes in slums in SSA. The interventions encompassed a variety of designs including pre-post quasi- experimental designs, interventions with matched comparison groups, community interventions without comparison or control groups and randomized controlled trials (Table 7). Most of these interventions were conducted in slum areas in East Africa with limited work in Southern Africa and West Africa. Since they targeted adolescents in slum settlement most addressed the socio-economic correlates of poor adolescent SRHR outcomes by building adolescents’ economic and social assets [27, 31, 47, 53–55]. The interventions for economic empowerment of girls included provision of microfinance, financial literacy, cash transfers, and savings programs aimed at reducing adolescents’ vulnerability to adverse SRHR outcomes. Some interventions also included the creation of safe spaces where girls could meet and receive training on various issues.

**Table 7:** Programs and interventions targeting adolescent SRHR

Name of the Intervention	City	Target	Study Design	Brief Description
Adolescent Girls Initiative-Kenya (AGI-K) (Austrian et al. 2015; Austrian et al.; 2018)	Nairobi	11–14 years girls	Randomized trial	Building adolescent girls assets (education, health, and wealth creation); cash transfer, savings, financial education, SRHR education and violence prevention
Binti Pamoja Centre (Daughters United Centre) (Carolina for Kibera 2007)	Nairobi	11–18 year girls	Community Intervention	Gender empowerment and creation of safe spaces for young people in order to 1) reduce violence, female genital mutilation, sexual abuse, rape, prostitution, and poverty; and 2) increase reproductive health knowledge, financial education, leadership and personal skills
BiruhTefta-Bright Future (Erulkar et al. 2013)	Addis Ababa	10–19 years girls	Quasi-experimental	Addresses social isolation by building social capital, literacy, providing information on HIV, reproductive health and GBV
CHANGE (Khoza et al. 2018)	Johannesburg	16–18 girls and boys	Randomized controlled trial	Examines the effects of unconditional versus conditional cash transfers on clinic and school attendance for HIV prevention
TRY-Tap and Reposition Youth (Hall, Dondo, and Sebstad 2006)	Nairobi	16–22 years young women	Intervention study with matched comparison	Improve livelihoods through microfinance, life skills, financial literacy in order to reduce vulnerability to adverse SRHR outcomes
Stepping Stones (Gibbs et al. 2017)	Durban	18-30 years (youth)	Cluster Randomized Control Trial	Comprehensive sexuality and behavior change communication (sexual health knowledge, communication skills, critical reflection and reduce sexual health risk)
Tupange (URHI-Urban Reproductive Health Initiative (Speizer et al. 2013)	Nairobi	10-24 years (young people)	Community Intervention study	Building capacity of service providers, contraceptive commodity security, demand-promotion and advocacy (dispel myth and misconception about contraceptives)
Virtual support group (Khaya HIV Positive) (Henwood et al. 2016)	Cape Town	12-25 years (young people)	Mhealth (social media) intervention study	Virtual support group. The chat-room used the MXit social networking platform to provide information on a youth-friendly HIV services (testing, treatment and care) and contraception

Some interventions focused on comprehensive sexuality education and behavior change communication strategies. These included information provision through mass media, social media, virtual space, social mobilization, advocacy, and through participatory activities [54, 56] . Others combined sexuality education with microcredit and community mobilization or gender empowerment training combined with life skills and financial literacy [27, 47]. The reported outcomes of these interventions included increased knowledge of HIV/AIDS and condom use [27, 57], reduction in the number of sexual partners [31, 55] reduced GBV [53, 55, 57] and transactional sexual relationships and improved gender attitudes [47, 57].

## Discussion

This scoping review mapped existing evidence on adolescent SRHR in slums in SSA and identified 54 studies published between January 2000 and May 2019. The review showed that adolescents and young people growing up in slums face tremendous challenges in relation to their SRHR needs resulting in poor outcomes such as early pregnancy, STIs, and sexual violence. Of the 54 studies identified, majority were conducted in slum areas in Nairobi, Kenya with very few in other slums areas across SSA.

The scope of the studies is also limited. The literature was skewed towards sexual behavior and HIV/AIDS with very few studies examining other aspects of SRHR such as contraception, abortion, GBV and STIs. The surge in the interest on adolescent sexual behavior is mainly related to the HIV/AIDS prevention programs [58]. This partly explains the narrow focus of these studies on “risky” sexual behavior to the exclusion of other facets of sexuality such as sexual satisfaction, sexual pleasure, eroticism and sexual identity. There is need for studies to explore the whole purview of sexuality such as sexual pleasure and satisfaction, sexual identity, orientation, sexual practices in addition to risky sexual behavior. Unexpectedly, there is minimal research on the utilization of maternal health services by adolescents overall despite the high risk of adolescent mothers to maternal morbidity and mortality due to their unique biological, sociological and economic status [59].

Since most studies were observational, cross-sectional quantitative studies, causal inferences cannot be made. Further, the relative dearth of qualitative studies that interrogate and give deeper insights to the experiences and needs of individuals mean that most studies provide limited data and/or information. As sexuality and other SRHR outcomes are partly driven by cultural norms and beliefs, qualitative studies exploring the roles/influence of contextual characteristics are needed [32, 60]. There is also need for studies to go beyond the conventional cross-sectional designs with a view to exploring how sexual behavior, identity, orientation, sexual practices and other SRHR behavior evolve especially in adolescence and younger ages. Studies interrogating puberty, romantic relationships and the effect of gender norms on romantic relationships are also needed. Mixed method designs that incorporate qualitative techniques can be undertaken to provide deeper understanding about these aspects of adolescent SRHR.

Our review highlighted age-overlaps in many of the studies identified. Although adolescent is normally defined as the period between 10 and 19 years [61], many studies identified did not strictly adhere to this definition. Barely half of the studies focused exclusively on adolescents (10–19), while the remaining combined adolescents with other groups namely; young people (10–24 years); youths 15–24 and reproductive age women and men (15–49 years). There is need for more studies on SRHR that focus exclusively on adolescent girls and boys in slums in SSA. Even very critical is the limited number of studies on very young adolescents (10–14), a group with what is called a ‘window of opportunity’ to address SRHR problems [62] .Early adolescence is a critical time to lay the foundation for healthy and fulfilling sexual and reproductive lives of adolescents. There is need for studies that categorize the adolescent period into early (10–14) and late (15–19) to allow for the comparison of SRHR outcomes and drivers between early versus late adolescence.

The results suggest a general paucity of intervention research that is focused on adolescent SRHR in the slum areas in the SSA. Specifically, intervention studies that allow for the comparison of impacts between early versus late adolescence are lacking. Intervention studies that address differential vulnerability in early and late adolescence among adolescent girls and boys in urban slums also are needed. Understanding what works to improve SRHR in early adolescence will likely lead to healthy trajectories across their life course [46, 63]. Although the identified interventions aimed at addressing the economic and social drivers of adolescent risk to poor SRHR outcomes, not all socio-structural factors highlighted in the review such as parenting were addressed by interventions. There are no interventions that focused on parenting as a key mechanism of socialization of adolescents, yet this was one of the factors that affect their healthy transition into adulthood [33, 34]. There is therefore need for interventions with and explicit focus on improving parent-child relationships (communication and other dimensions of parenting) in the slums. Finally, comparative studies across sites and SRHR components are lacking. There is a dearth of multi-country or multi-site and multi-theme studies on adolescent SRHR in slums settlement in SSA that can provide generalizable evidence.

Understanding the unique challenges of urban poverty is critical in understanding the drivers of adolescent SRHR outcomes in the slum settings. Rapid urbanization is taking place in SSA in the context of poor economic performance, which presents challenges for adolescents in cities [8, 64]. There are wide disparities in health and socio-economic outcomes between the wealthy and poor urban adolescents: more adolescents in poor urban settings engage in riskier sexual behaviors, have higher HIV infection, experience higher mortality and are more likely to experience violence and drug abuse than their wealthier counterparts [16, 65, 66]. The review focused on several key SRHR issues (sexual behavior, adolescent pregnancy, HIV/AIDS, and GBV among others) but yet has not included studies around puberty, romantic relationships and the effect of gender norms on romantic relationships.

To our knowledge, this is the first scoping review examining adolescent SRHR in urban slums in SSA. Although we used rigorous and transparent methods to ensure a comprehensive search of the literature, it is possible that we did not retrieve all studies in peer-reviewed journals or grey literature. Further, our review only included articles published in English. Nonetheless, the review has yielded important findings on several key SRHR issues that affect adolescents living in resource-limited urban slums in SSA, a region with a high burden of poor SRHR outcomes including early and unintended pregnancy, HIV/AIDS, and GBV.

## Conclusion

The results of this review point to several potential target areas for programming and research aimed at improving adolescents’ SRHR. To improve and promote adolescent SRHR, development and well-being, it is important to understand the broader socio-ecological context of slum residence as a risky environment with poor, unhealthy and unsafe living conditions, rather than focusing solely on individual SRHR-related behaviour. Understanding and addressing adolescents’ SRHR requires a comprehensive understanding of the contexts that increase their vulnerability to poor outcomes. Interventions that address this risky environment will help adolescents transition into adulthood as a healthy workforce that can help countries in SSA achieve a demographic dividend.

## Data Availability

The list of publications used for the scoping review are available in the manuscript and can also be made available upon request.

## Abbreviations

AIDS: Acquired immunodeficiency syndrome
GBV: Gender-based violence
HIV: Human immunodeficiency virus
LMICs: Low- and Middle-Income Countries
SRHR: Sexual and Reproductive Health and Rights
SSA: Sub-Saharan Africa
STI: Sexually transmitted infection
UNFPA: United Nations Population Fund
WHO: World Health Organization
